# Approximate Analytic Expression for the Time-Dependent Transient Electrophoretic Mobility of a Spherical Colloidal Particle

**DOI:** 10.3390/molecules27165108

**Published:** 2022-08-11

**Authors:** Hiroyuki Ohshima

**Affiliations:** Faculty of Pharmaceutical Sciences, Tokyo University of Science, 2641 Yamazaki, Noda, Chiba 278-8510, Japan; ohshima@rs.noda.tus.ac.jp

**Keywords:** transient electrophoresis, transient electrophoretic mobility, spherical colloidal particle, step electric field

## Abstract

The general expression is derived for the Laplace transform of the time-dependent transient electrophoretic mobility (with respect to time) of a spherical colloidal particle when a step electric field is applied. The transient electrophoretic mobility can be obtained by the numerical inverse Laplace transformation method. The obtained expression is applicable for arbitrary particle zeta potential and arbitrary thickness of the electrical double layer around the particle. For the low potential case, this expression gives the result obtained by Huang and Keh. On the basis of the obtained general expression for the Laplace transform of the transient electrophoretic mobility, we present an approximation method to avoid the numerical inverse Laplace transformation and derive a simple approximate analytic mobility expression for a weakly charged particle without involving numerical inverse Laplace transformations. The transient electrophoretic mobility can be obtained directly from this approximate mobility expression without recourse to the numerical inverse Laplace transformation. The results are found to be in excellent agreement with the exact numerical results obtained by Huang and Keh.

## 1. Introduction

Standard theories of electrokinetics, including electrophoresis, consider the motion of colloidal particles under an applied static electric field, while only a few studies treat time-dependent transient electrophoresis, that is, the electrophoretic motion of a colloidal particle under an applied step electric field [1,2,3,4,5,6,7,8,9,10,11,12,13,14,15,16]. The theory of transient electrophoresis was initiated by Morison [1,2] and Ivory [3,4] and has been advanced significantly by Keh and his coworkers [5,6,7,9,10,13,14,15]. In particular, for a weakly charged spherical particle, Huang and Keh [7] have derived an exact expression for the Laplace transform (with respect to time) of the transient electrophoretic mobility of the particle. The transient electrophoretic mobility of the particle can then be obtained by the numerical inverse Laplace transformation method. This method, however, which requires tedious numerical calculations, is not very convenient for practical uses. In the present paper, we derive the general expression for the Laplace transform of the transient electrophoretic mobility of a spherical particle. We then propose an approximation method to avoid numerical inverse Laplace transformations and derive a simple analytic mobility expression for a weakly charged spherical particle without involving the numerical inverse Laplace transformation.

## 2. Theory

**Figure 1:**
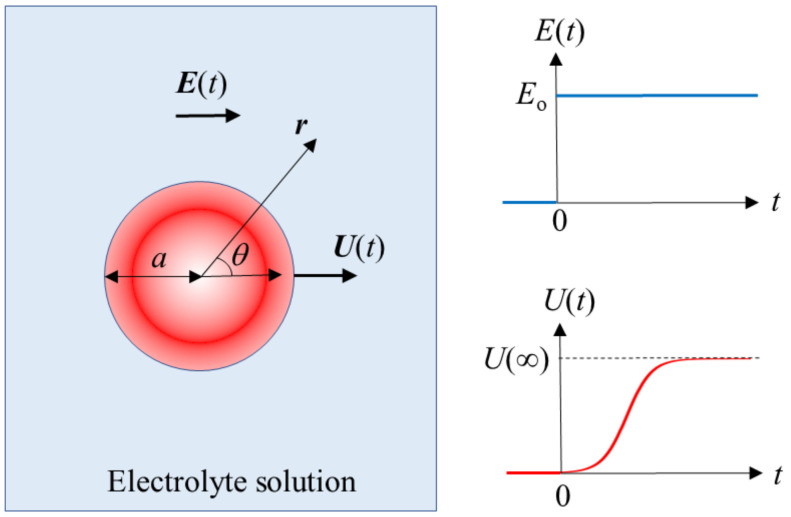
Spherical colloidal particle of radius *a* and zeta potential *ζ* moving with transient electrophoretic velocity ***U***(*t*) in an electrolyte solution under an applied step electric field ***E***(*t*). *U*(∞) is the magnitude of the static electrophoretic velocity at *t* = ∞.

Consider a spherical colloidal particle of radius *a*, mass density *ρ*_p_, and zeta potential *ζ* in an aqueous liquid of relative permittivity *ε*_r_, mass density *ρ*_o_, and viscosity *η* that contains a general electrolyte consisting of *N* ionic species of valence z*_i_*, bulk concentration (number density) *n_i_*^∞^, and drag coefficient *λ_i_* (*i* = 1, 2, …, *N*) (Figure 1). Suppose that at time *t* = 0, a step electric field ***E***(*t*) is applied to the particle, viz.,

(1) $$E\left(t\right)=\left\{\begin{array}{cc} 0, & t=0\\ E_{o}, & t>0 \end{array}\right.$$

where ***E***_o_ is constant and the particle starts to move with an electrophoretic velocity ***U***(*t*) in the direction parallel to ***E***_o_. The transient electrophoretic mobility *μ*(*t*) of the particle is defined by ***U***(*t*) = *μ*(*t*)***E***(*t*) = *μ*(*t*)***E***_o_. The origin of the spherical coordinate system (*r*, *θ*, *φ*) is held fixed at the center of the particle and the polar axis (*θ* = 0) is set parallel to ***E***_o_. We treat the case where the following conditions are satisfied: (i) The liquid can be regarded as incompressible. (ii) The applied electric field ***E***(*t*) is weak so that the particle velocity ***U***(*t*) is proportional to ***E***(*t*), and terms involving the square of the liquid velocity in the Navier–Stokes equation can be neglected. (iii) The slipping plane (at which the liquid velocity ***u***(***r***, *t*) relative to the particle is zero) is located on the particle surface (at *r* = *a*). (iv) Electrolyte ions cannot penetrate the particle surface. (v) In the absence of ***E***(*t*), the equilibrium ion distribution obeys the Boltzmann distribution, and the equilibrium electric potential is described by the Poisson–Boltzmann equation.

Under these conditions (1)–(v), the fundamental electrokinetic equations for the liquid flow velocity ***u***(***r***, *t*) at position ***r***(*r*, *θ*, *φ*) and time *t* and the velocity ***v****_i_*(***r***, *t*) of *i* th ionic species—which are similar to those for the dynamic electrophoresis of a spherical particle in an applied oscillating electric field [17]—are given by

(2) $$\rho_{o}\frac{\partial }{\partial t}\left\{u\left(r,t\right)+U\left(t\right)\right\}+\eta \nabla \times \nabla \times u\left(r,t\right)+\nabla p\left(r,t\right)+\rho_{\mathrm{el}}\left(r,t\right)\nabla \psi \left(r,t\right)=0$$

(3) $$\nabla \cdot u\left(r,t\right)=0$$

(4) $$v_{i}\left(r,t\right)=u\left(r,t\right)-\frac{1}{\lambda_{i}}\nabla \mu_{i}\left(r,t\right)$$

(5) $$\frac{\partial n_{i}\left(r,t\right)}{\partial t}+\nabla \cdot \left\{n_{i}\left(r,t\right)v_{i}\left(r,t\right)\right\}=0$$

with

(6) $$\rho_{\mathrm{el}}\left(r,t\right)=\overset{N}{\underset{i=1}{\sum }}z_{i}n_{i}\left(r,t\right)$$

(7) $$\mu_{i}\left(r,t\right)=\mu_{i}^{o}+z_{i}e\psi \left(r,t\right)+kT\ln\left[n_{i}\left(r,t\right)\right]$$

(8) $$\Delta \psi \left(r,t\right)=-\frac{\rho_{\mathrm{el}}\left(r,t\right)}{\varepsilon_{r}\varepsilon_{o}}$$

where *e* is the elementary electric charge, *k* is the Boltzmann constant, *T* is the absolute temperature, *ε*_o_ is the permittivity of a vacuum, *p*(***r***, *t*) is the pressure, *ρ*_el_(***r***, *t*) is the charge density, and *ψ*(***r***, *t*) is the electric potential. Equations (2) and (3) are the Navier–Stokes equation and the equation of continuity for an incompressible flow (condition (i)), where the term *ρ*_o_(***u***·∇)***u*** has been omitted (condition (ii)). The term involving the particle velocity ***U*** (*t*) in Equation (2) arises from the fact that the particle has been chosen as the frame of reference for the coordinate system. Equation (4) expresses that the flow ***v**_i_*(***r***, *t*) of the *i* th ionic species is caused by the liquid flow ***u***(***r***, *t*) and the gradient of the electrochemical potential *μ_i_*(***r***, *t*), given by Equation (7), in which $\mu_{i}^{o}$ is a constant term. Equation (5) is the continuity equation for the *i* th ionic species of concentration *n_i_*(***r***, *t*). Equation (8) is the Poisson equation.

The following boundary conditions at the particle surface (at *r* = *a*) and far from the particle (*r* → ∞) must be satisfied:

(9) $$u\left(r,t\right)=0\;\mathrm{at}\;\;t=0$$

(10) $$u\left(r,t\right)=0\;\mathrm{at}\;r=a$$

(11) $$u\left(r,t\right)\to -U\left(r,t\right)+\frac{a^{3}\left(\rho_{p}-\rho_{o}\right)}{3\rho_{o}r^{3}}\;\left[U\left(r,t\right)-3\left\{\left(U\left(r,t\right)\cdot \hat{r}\right)\hat{r}\right\}\right]\;\mathrm{as}\;r\to \infty$$

(12) $$v_{i}\left(r,t\right)\cdot \hat{n}=0\;\;\mathrm{at}\;r=a$$

(13) $$\psi \left(r,t\right)=-E\left(t\right)\cdot r\;\mathrm{as}\;r\to \infty$$

where $\hat{r}$ = ***r***/*r* (*r* = |***r***|) and $\hat{n}$ is the unit normal outward from the particle surface. Equation (10) states that the slipping plane (at which ***u***(***r***, *t*) = **0**) is located on the particle surface (condition (iii)). Equation (11) can be derived from the equation of motion of the particle, as in the case of dynamic electrophoresis [17]. Equation (12) follows from condition (iv). Equation (13) follows from the fact that the electric potential *ψ*(***r****, t*) tends to the potential of the applied electric field ***E***(*t*) as *r* →∞.

For a weak field ***E***(*t*), the deviations of *n_j_*(***r****, t*), *ψ*(***r****, t*), and *μ_j_*(***r****, t*) from their equilibrium values (i.e., those in the absence of ***E***(*t*)) due to the applied field ***E***(*t*) are small. In this situation, we may write

(14) $$n_{i}\left(r,t\right)=n_{i}^{\left(0\right)}\left(r\right)+\delta n_{i}\left(r,t\right)$$

(15) $$\psi \left(r,t\right)=\psi^{\left(0\right)}\left(r\right)+\delta \psi \left(r,t\right)$$

(16) $$\mu_{i}\left(r,t\right)=\mu_{i}^{\left(0\right)}+\delta \mu_{i}\left(r,t\right)$$

where the quantities with superscript (0) refer to those at equilibrium and $\mu_{i}^{\left(0\right)}$ is a constant independent of *r*. We assume that the equilibrium concentration $n_{i}^{\left(0\right)}\left(r\right)$ obeys the Boltzmann distribution and the equilibrium electric potential satisfies the Poisson–Boltzmann equation (condition (v)), viz.

(17) $$n_{i}^{\left(0\right)}\left(r\right)=n_{i}^{\infty }\left(r\right)e^{-z_{i}y\left(r\right)}$$

(18) $$\Delta y\left(r\right)=-\kappa^{2}\frac{\sum_{i=1}^{N}z_{i}n_{i}^{\infty }e^{-z_{i}y\left(r\right)}}{\sum_{i=1}^{N}z_{i}^{2}n_{i}^{\infty }}$$

with

(19) $$y\left(r\right)=\frac{e\psi^{\left(0\right)}\left(r\right)}{kT}$$

(20) $$\kappa =\sqrt{\frac{e^{2}}{\varepsilon_{r}\varepsilon_{o}kT}\overset{N}{\underset{i=1}{\sum }}z_{i}^{2}n_{i}^{\infty }}$$

where *y*(*r*) is the scaled equilibrium electric potential, and *κ* is the Debye–Hückel parameter (1/*κ* is the Debye length).

By substituting Equations (14)–(16) into Equation (2), and neglecting the products of the small quantities, we obtain

(21) $$\rho_{o}\frac{\partial }{\partial t}\nabla \times u\left(r,t\right)+\eta \nabla \times \nabla \times \nabla \times u\left(r,t\right)=\overset{N}{\underset{i=1}{\sum }}\nabla \delta \mu_{i}\left(r,t\right)\times \nabla n_{i}^{\left(0\right)}\left(r\right)$$

and form Equations (4) and (5)

(22) $$\frac{\partial }{\partial t}\delta n_{i}\left(r,t\right)+\nabla \cdot \left\{n_{i}^{\left(0\right)}\left(r\right)u\left(r,t\right)-\frac{1}{\lambda_{i}}n_{i}^{\left(0\right)}\left(r\right)\nabla \delta \mu_{i}\left(r,t\right)\right\}=0$$

Furthermore, symmetry considerations permit us to write

(23) $$u\left(r,t\right)=\left(-\frac{2}{r}h\left(r,t\right)E\left(t\right)\cos\theta ,\;\frac{1}{r}\frac{d}{dr}\left(rh\left(r,t\right)\right)E\left(t\right)\sin\theta ,\;0\right)$$

(24) $$\delta \mu_{i}\left(r,\;t\right)=-z_{i}e\phi_{i}\left(r,t\right)E\left(t\right)\cos\theta$$

(25) $$\delta \psi \left(r,\;t\right)=-Y\left(r,t\right)E\left(t\right)\cos\theta$$

where ***E***(*t*) is the magnitude of ***E***(*t*), and *h*(*r, t*), *ϕ_i_*(*r*, *t*), and *Y*(*r*, *t*) are functions of *r* and *t*. By substituting Equations (23)–(25) into Equations (21) and (22), we obtain the following equations for *h*(*r*), *ϕ_i_*(r), and *Y*(*r*):

(26) $$L\left[Lh\left(r,t\right)-\frac{1}{\nu }\frac{\partial h\left(r,t\right)}{\partial t}\right]=G\left(r,t\right)$$

(27) $$L\phi_{i}\left(r,t\right)-\frac{\lambda_{i}}{kT}\frac{\partial }{\partial t}\left\{\phi_{i}\left(r,t\right)-Y\left(r,t\right)\right\}=\frac{dy\left(r\right)}{dr}\left\{z_{i}\frac{d\phi_{i}\left(r,t\right)}{dr}-\frac{2\lambda_{i}}{e}\frac{h\left(r,t\right)}{r}\right\}$$

(28) $$LY\left(r,t\right)=\frac{1}{\varepsilon_{r}\varepsilon_{o}kT}\overset{N}{\underset{i=1}{\sum }}z_{i}^{2}e^{2}n_{i}^{\left(0\right)}\left(r\right)\left\{Y\left(r,t\right)-\phi_{i}\left(r,t\right)\right\}$$

where

(29) $$L=\frac{d}{dr}\frac{1}{r^{2}}\frac{d}{dr}r^{2}=\;\frac{d^{2}}{dr^{2}}+\frac{2}{r}\;\frac{d}{dr}-\frac{2}{r^{2}}$$

is a differential operator. *G*(*r*, *t*) is defined by

(30) $$G\left(r,t\right)=-\frac{e}{\eta r}\frac{dy}{dr}\overset{N}{\underset{i=1}{\sum }}z_{i}^{2}n_{i}^{\infty }e^{-z_{i}y}\phi_{i}\left(r,t\right)$$

and

(31) $$\nu =\frac{\eta }{\rho_{o}}$$

is the kinematic viscosity. The boundary conditions (Equations (9)*–*(13)) are rewritten as those for *h(r, t)*, *ϕ_i_(r, t)*, and *Y(r, t)*, as follows:

(32) $$h\left(r,\;t\right)=\frac{dh\left(r,\;t\right)}{dr}=0\;\;\mathrm{at}\;\;t=0$$

(33) $$h\left(r,\;t\right)=\frac{dh\left(r,\;t\right)}{dr}=0\;\;\mathrm{at}\;r=a$$

(34) $$h\left(r,t\right)\to \frac{\mu \left(t\right)}{2}r+\frac{a^{3}\left(\rho_{p}-\rho_{o}\right)}{3\rho_{o}r^{2}}\;\mu \left(t\right)\;\;\mathrm{as}\;r\to \infty$$

(35) $$\phi_{i}\left(r,t\right)\to r\;\mathrm{as}\;r\to \infty$$

(36) $$\frac{\phi_{i}\left(r,t\right)}{dr}=0\;\;\mathrm{at}\;\;r=a$$

(37) $$Y\left(r,t\right)\to r\;\mathrm{as}\;r\to \infty$$

The transient electrophoretic mobility *μ*(*t*) can be obtained from Equation (34), viz.,

(38) $$\mu \left(t\right)=\frac{U\left(t\right)}{E\left(t\right)}=\frac{U\left(t\right)}{E_{o}}=2\underset{r\to \infty }{\lim}\frac{h\left(r,\;t\right)}{r}$$

Here, *h*(*r*, *t*) is the solution to Equation (26), which is most easily solved by using the Laplace transformation with respect to time *t*. The Laplace transforms $\hat{h}\left(r,s\right)$, $\hat{G}\left(r,s\right)$, and $\hat{\mu }\left(s\right)$ of *h*(*r*, *t*), *G*(*r*, *t*), and *μ*(*t*), respectively, are given by,

(39) $$\hat{h}\left(r,s\right)=\int_{0}^{\infty }h\left(r,t\right)e^{-st}dt$$

(40) $$\hat{G}\left(r,s\right)=\int_{0}^{\infty }G\left(r,t\right)e^{-st}dt$$

(41) $$\hat{\mu }\left(s\right)=\int_{0}^{\infty }\mu \left(t\right)e^{-st}dt$$

and the Laplace transform of Equation (38) is

(42) $$\hat{\mu }\left(s\right)=2\underset{r\to \infty }{\lim}\frac{\hat{h}\left(r,s\right)}{r}$$

Thus, the Laplace transform of Equation (26) gives

(43) $$L\left[L\hat{h}\left(r,s\right)-\frac{s}{\nu }\hat{h}\left(r,s\right)\right]=\hat{G}\left(r,s\right)$$

By solving Equation (43) and using Equation (40), we obtain the following general expression for $\hat{\mu }\left(s\right)$:

(44) $$\hat{\mu }\left(s\right)=-\frac{2\nu \int_{a}^{\infty }\left\{1+a\sqrt{\frac{s}{\nu }}+\frac{a^{2}s}{3\nu }-\left(1+\sqrt{\frac{s}{\nu }}\;r\right)\exp\left[-\sqrt{\frac{s}{\nu }}\;\left(r-a\right)\right]-\frac{sr^{3}}{3\nu a}\right\}\hat{G}\left(r,s\right)dr\;}{3s\left\{1+a\sqrt{\frac{s}{\nu }}+\frac{a^{2}s}{3\nu }+\frac{2a^{2}\left(\rho_{p}-\rho_{o}\right)s}{9\nu \rho_{o}}\right\}}$$

The transient electrophoretic mobility *μ*(*t*) can be obtained by the numerical inverse transform of Equation (44).

## 3. Results and Discussion

Equation (44) is the required expression for $\hat{\mu }\left(s\right)$. The transient electrophoretic mobility *μ*(*t*) can be obtained from Equation (44) by the numerical inverse transform method.

Now, consider the low *ζ* potential case. In this case, Equations (27) and (28) yield

(45) $$\phi_{i}\left(r,t\right)=Y\left(r,t\right)=r+\frac{a^{3}}{2r^{2}}$$

Then, Equation (30) becomes

(46) $$G\left(r,t\right)=-\frac{\varepsilon_{r}\varepsilon_{o}\kappa^{2}}{\eta }\left(1+\frac{a^{3}}{2r^{3}}\right)\frac{d\psi^{\left(0\right)}\left(r\right)}{dr}$$

The Laplace transform $\hat{G}\left(r,s\right)$ of *G*(*r*, *t*) is given by

(47) $$\hat{G}\left(r,s\right)=\frac{G\left(r,t\right)}{s}=-\frac{\varepsilon_{r}\varepsilon_{o}\kappa^{2}}{\eta s}\left(1+\frac{a^{3}}{2r^{3}}\right)\frac{d\psi^{\left(0\right)}\left(r\right)}{dr}$$

where the equilibrium electric potential *ψ*^(0)^(*r*) for the low *ζ* potential case is given by

(48) $$\psi^{\left(0\right)}\left(r\right)=\zeta \frac{a}{r}e^{-\kappa (r-a})$$

which is obtained from the linearized Poisson–Boltzmann equation ∆*ψ*^(0)^(*r*)=*κ*^2^*ψ*^(0)^(*r*) (see Equation (18)). By substituting Equation (47) into Equation (44), we obtain

(49) $$\hat{\mu }\left(s\right)=\frac{2\varepsilon_{r}\varepsilon_{o}\kappa^{2}\nu \int_{a}^{\infty }\left\{1+a\sqrt{\frac{s}{\nu }}+\frac{a^{2}s}{3\nu }-\left(1+\sqrt{\frac{s}{\nu }}\;r\right)\exp\left[-\sqrt{\frac{s}{\nu }}\;\left(r-a\right)\right]-\frac{sr^{3}}{3\nu a}\right\}\left(1+\frac{a^{3}}{2r^{3}}\right)\frac{d\psi^{\left(0\right)}\left(r\right)}{dr}dr\;}{3\eta s^{2}\left\{1+a\sqrt{\frac{s}{\nu }}+\frac{a^{2}s}{3\nu }+\frac{2a^{2}\left(\rho_{p}-\rho_{o}\right)s}{9\nu \rho_{o}}\right\}}$$

which agrees with Huang and Keh’s result (Equation (31) in ref. [7]). Huang and Keh [7] obtained the transient electrophoretic mobility *μ*(*t*) by using the numerical inverse transform of Equation (49). This method, however, involves tedious numerical calculations and is not very convenient for practical uses. The reason for this is that the integration in Equation (49) cannot be carried out analytically due to the presence of the factor 1 + *a*^3^/2*r*^3^. In order to avoid this difficulty, we employ the same approximation method as used for the static electrophoresis problem [18]. We first note that the integrand in Equation (49) has a sharp maximum around *r* = *a* + *δ*/*κ*, *δ* being a factor of order unity. This is because the electrical double layer (of thickness 1/*κ*) around the particle is confined in the narrow region between *r* = *a* and *r* ≈ *a* + 1/*κ*. Since the factor (1 + *a*^3^/2*r*^3^) in the integrand of Equation (49) varies slowly with *r* as compared with the other factors, one may approximately replace *r* in the factor (1 + *a*^3^/2*r*^3^) by *r* = *a* + *δ*/*κ* and take it out before the integral sign, viz.,

(50) $$1+\frac{a^{3}}{2r^{3}}\approx 1+\frac{1}{2{\left(1+\frac{\delta }{\kappa a}\right)}^{3}}$$

In a similar problem in static electrophoresis [18], we have found that the best approximation can be achieved if *δ* is chosen to be 2.5/$\left(1+2e^{-\kappa a}\right)$. We use this choice of *δ* in the transient electrophoresis problem. By using this approximation, the integration in Equation (49) can be carried out analytically to give

(51) $$\hat{\mu }\left(s\right)=\frac{1+\left(1+\frac{1}{\kappa a}\right)a\sqrt{\frac{s}{\nu }}}{\left\{1+a\sqrt{\frac{s}{\nu }}+\frac{a^{2}s}{3\nu }+\frac{2a^{2}\left(\rho_{p}-\rho_{o}\right)s}{9\nu \rho_{o}}\right\}\left(1+\frac{1}{\kappa }\sqrt{\frac{s}{\nu }}\right)}\frac{\mu_{H}}{s}$$

with

(52) $$\mu_{H}=\frac{2\varepsilon_{r}\varepsilon_{o}\zeta }{3\eta }\left[1+\frac{1}{2{\left\{1+\frac{2.5}{\kappa a\left(1+2e^{-\kappa a}\right)}\right\}}^{3}}\right]$$

Equation (52) has been found to be an excellent approximate expression for the exact expression of Henry’s static mobility formula [18], viz.,

(53) $$\mu_{H}=\frac{\varepsilon_{r}\varepsilon_{o}\zeta }{\eta }\left\{1-\left\{5e^{\kappa a}E_{7}\left(\kappa a\right)-2e^{\kappa a}E_{5}\left(\kappa a\right)\right\}\right\}$$

where

(54) $$E_{n}\left(\kappa a\right)={\left(\kappa a\right)}^{n-1}\int_{\kappa a}^{\infty }\frac{e^{-x}}{x^{n}}dx$$

is the exponential integral of order *n*. Note that in the limit of *t* →∞, *μ*(*t*) approaches the static electrophoretic mobility *μ*_H_ and Equation (53) can be derived directly from Equation (49), viz.,

(55) $$\mu \left(\infty \right)=\underset{s\to 0}{\lim}s\hat{\mu }\left(s\right)=-\frac{\varepsilon_{r}\varepsilon_{o}{\left(\kappa a\right)}^{2}}{9\eta }\int_{a}^{\infty }\left(1-\frac{3r^{2}}{a^{2}}+\frac{2r^{3}}{a^{3}}\right)\left(1+\frac{a^{3}}{2r^{3}}\right)\frac{d\psi^{\left(0\right)}\left(r\right)}{dr}dr$$

which yields Equation (53), that is, *μ*(∞) = *μ*_H_.

The transient electrophoretic mobility *μ*(*t*) can now be obtained analytically from Equation (51) without recourse to the numerical inverse Laplace transformation. The result is

(56) $$\mu \left(t\right)=\left\{\;\frac{\left(\kappa a-q_{1}-\kappa aq_{1}\right)q_{2}}{\left(\kappa a-q_{1}\right)\left(q_{2}-q_{1}\right)}M\left(\frac{q_{1}\sqrt{\nu t}}{a}\right)-\frac{\left(\kappa a-q_{2}-\kappa aq_{2}\right)q_{1}}{\left(\kappa a-q_{2}\right)\left(q_{2}-q_{1}\right)}M\left(\frac{q_{2}\sqrt{\nu t}}{a}\right)-\frac{\kappa aq_{1}q_{2}M\left(\kappa \sqrt{\nu t}\right)}{\left(\kappa a-q_{1}\right)\left(\kappa a-q_{2}\right)}\right\}\mu_{H}$$

with

(57) $$q_{1}=\frac{9}{2\left(1+\frac{2\rho_{p}}{\rho_{o}}\right)}\left(1+\frac{1}{3}\sqrt{5-\frac{8\rho_{p}}{\rho_{o}}}\right)$$

(58) $$q_{2}=\frac{9}{2\left(1+\frac{2\rho_{p}}{\rho_{o}}\right)}\left(1-\frac{1}{3}\sqrt{5-\frac{8\rho_{p}}{\rho_{o}}}\right)$$

(59) $$M\left(z\right)=1-e^{z^{2}}\mathrm{erfc}\left(z\right)$$

where *q*_1_ and *q*_2_ may be either real or complex (*μ*(*t*) is always real), and erfc(*z*) is the complementary error function, defined by

(60) $$\mathrm{erfc}\left(z\right)=\frac{2}{\sqrt{\pi }}\int_{z}^{\infty }e^{-x^{2}}dx$$

and *M*(*z*) tends to 0 as *z* → 0 and to 1 as *z* → ∞.

The transient electrophoretic mobility *μ*(*t*) given by Equation (56) tends to the correct limiting mobilities for large *κa* and small *κa*. In the limit of large *κa* (*κa* » 1), Equation (56) tends to

(61) $$\mu \left(t\right)=\frac{\varepsilon_{r}\varepsilon_{o}\zeta }{\eta }\left\{\;\frac{\left(1-q_{1}\right)q_{2}}{q_{2}-q_{1}}M\left(\frac{q_{1}\sqrt{\nu t}}{a}\right)-\frac{\left(1-q_{2}\right)q_{1}}{q_{2}-q_{1}}M\left(\frac{q_{2}\sqrt{\nu t}}{a}\right)\right\}$$

which agrees with Morrison’s result (Equation (32) in ref. [1]). In the opposite limit of small *κa* (*κa* « 1), Equation (55) tends to

(62) $$\mu \left(t\right)=\frac{2\varepsilon_{r}\varepsilon_{o}\zeta }{3\eta }\left\{\;\frac{q_{2}}{q_{2}-q_{1}}M\left(\frac{q_{1}\sqrt{\nu t}}{a}\right)-\frac{q_{1}}{q_{2}-q_{1}}M\left(\frac{q_{2}\sqrt{\nu t}}{a}\right)\right\}$$

which agrees with Keh and Huang’s result (Equation (44) in ref. [6]).

**Figure 2:**
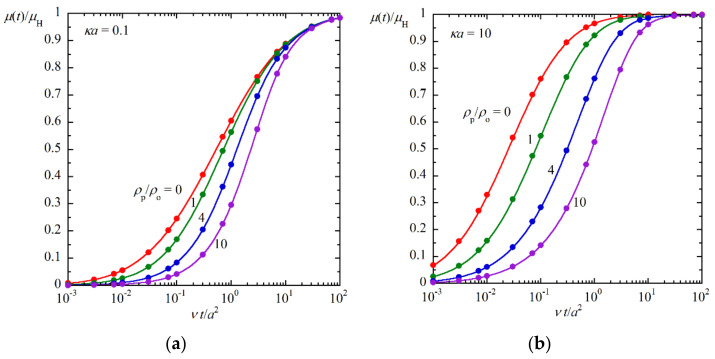
Time-dependent transient electrophoretic mobility *μ*(*t*) of a spherical colloidal particle of radius *a* and mass density *ρ*_p_ in an electrolyte solution of the Debye length 1/*κ*, mass density *ρ*_o_, and viscosity *η*. The ratio of *μ*(*t*) at time *t* to its value *μ*(∞) at *t* = ∞, which is equal to Henry’s static mobility formula *μ*_H_ ((*μ*(∞) = *μ*_H_), is plotted as a function of the scaled time *νt*/*a*^2^, *ν* being the kinematic viscosity ((*ν* = *η*/*ρ*_o_). Solid lines are approximate results calculated with Equation (56) for *ρ*_p_/*ρ*_o_ = 0, 1, 4, and 10 at *κa* = 0.1 (**a**) and *κa* = 10 (**b**). The symbols (●) are the corresponding exact numerical results by Huang and Keh [7]. The present approximation (Equation (56)) is found to be in excellent agreement with the exact numerical results [7].

In order to see the accuracy of Equation (56), in Figure 2, we compare the approximate results calculated by Equation (56) with the exact numerical results obtained by Huang and Keh [7] for several values of the ratio *ρ*_p_/*ρ*_o_ for two values of *κa* (*κa* = 0.1 and 10). The agreement between the approximate results (Equation (56)) and the exact numerical results [7] is found to be excellent agreement with negligible errors. (Both results agree with each other within the line width.)

Finally, it should be mentioned that there is a simple correspondence between the Laplace transform $\hat{\mu }\left(s\right)$ of the transient electrophoretic mobility *μ*(*t*) and the dynamic electrophoretic mobility *μ*_D_ of a spherical particle under an oscillating electric field of frequency *ω* (see the original work by O’Brien [19] as well as ref. [17]). The dynamic mobility *μ*_D_ of a spherical particle of radius *a* is given by Equation (83) in ref. [17], viz.,

(63) $$\mu_{D}=\frac{2\int_{a}^{\infty }\left\{H\left(a\right)-H\left(r\right)\right\}G\left(r\right)dr\;}{3\gamma^{2}\left\{H\left(a\right)-\Gamma \right\}}$$

with

(64) $$H\left(r\right)=\left(1-i\gamma r\right)e^{i\gamma \left(r-a\right)}-\frac{\gamma^{2}r^{3}}{3a}$$

(65) $$\Gamma =\frac{2{\left(\gamma a\right)}^{2}\left(\rho_{p}-\rho_{o}\right)}{9\rho_{o}}$$

(66) $$\gamma =\sqrt{\frac{i\omega }{\nu }}=\left(1+i\right)\sqrt{\frac{\omega }{2\nu }}$$

where *G*(*r*) is the same as Equation (46). In Equation (63), by replacing -*iω* with *s* and *G*(*r*) by *G*(*r*)/*s*, then Equation (63) becomes Equation (49) (and thus Equation (51)).

## 4. Conclusions

We have derived the general expression, Equation (44), for the Laplace transform $\hat{\mu }\left(s\right)$ of the time-dependent transient electrophoretic mobility *μ*(*t*) of a spherical colloidal particle when a step electric field ***E***(*t*) (Equation (1)) is applied. The transient electrophoretic mobility *μ*(*t*) can be obtained by the numerical inverse Laplace transformation method [7], which, however, requires tedious numerical calculations. Equation (44) is applicable for arbitrary values of the particle zeta potential *ζ* and arbitrary values of the Debye length 1/*κ*. For the low *ζ* potential case, Equation (44) reduces to Equation (46), which agrees with the result obtained by Huang and Keh [7]. On the basis of Equation (49), we have derived a simple mobility expression, Equation (56), which does not involve the numerical inverse Laplace transformation. The approximate results obtained by Equation (59) are in excellent agreement with the exact numerical results of Huang and Keh [7].

## Data Availability

Not applicable.

## Sample Availability

Samples of the compounds are not available from the authors.
